# SOX17 restrains proliferation and tumor formation by down-regulating activity of the Wnt/β-catenin signaling pathway via trans-suppressing β-catenin in cervical cancer

**DOI:** 10.1038/s41419-018-0782-8

**Published:** 2018-07-03

**Authors:** Lu Li, Wen-Ting Yang, Peng-Sheng Zheng, Xiao-Fang Liu

**Affiliations:** 1 Department of Reproductive Medicine, The First Affiliated Hospital of Xi’an Jiaotong University, Shaanxi Xi’an, 710061 The People’s Republic of China; 2 Section of Cancer Stem Cell Research, Key Laboratory of Environment and Genes Related to Diseases, Ministry of Education of the People’s Republic of China, Shaanxi Xi’an, 710061 The People’s Republic of China

## Abstract

The SRY-box containing gene 17 (SOX17) is considered as a regulator in stemness maintenance and a suppressor in some malignant tumors. However, the biological function and molecular mechanism of SOX17 in the process of initiation and progression of cervical cancer remain obscure. In this study, immunohistochemistry showed that the expression of SOX17 was high in the normal cervix, moderate in the high-grade squamous intraepithelial lesion, and low in the cervical cancer. SOX17 inhibited the proliferation and viability of cervical cancer cells in vitro as well as tumor formation in vivo. Additionally, SOX17 induced the cell cycle arrest at the transition from the G_0_/G_1_ phase to the S phase. The TOP/ FOP-Flash reporter assay and Western blotting showed SOX17 inhibited the activity of the Wnt/β-catenin signaling pathway in cervical cancer. Further, firefly luciferase reporter assay and quantitative chromatin immunoprecipitation (qChIP) assays confirmed that SOX17 trans-suppressed the expression of β-catenin by directly binding to the specific region of the β-catenin promoter. Together, our data demonstrated that SOX17 restrained the proliferation and tumor formation by down-regulating the activity of the Wnt/β-catenin signaling pathway via trans-suppression of β-catenin in cervical cancer.

## Introduction

Cervical cancer is the fourth most common cancer in women and the seventh overall^1^. According to the latest authoritative data, there were estimated 527,600 new cervical cancer cases and 265,700 deaths worldwide in 2012^2^. Although high-risk human papillomavirus (HPV) is well established as the major risk factor for cervical cancer carcinogenesis^3^, most HPV infections are transient and cleared within months^4^. Furthermore, the genetic alterations and epigenetic modifications involved in the initiation and progression of cervical cancer have not been clearly elucidated yet^5^. Recently, extensive studies have shown that some stem cell self-renewal-associated transcription factors, such as SOX2^6^, SOX9^7^, NANOG^8^, KLF4^9^, LGR5^10^, UTF1^11^, OCT4^12^, and DAX1^13^, are anomaly modulated and functionally alter signaling pathways during cervical cancer carcinogenesis.

As a member of the SOX transcription factor family, SOX17 (SRY-box containing gene 17) has been considered a well-known endoderm marker^14^. SOX17 plays a key role in the generation and maintenance of neonatal hematopoietic stem cells (HSCs)^15^ as well as in regulating the fate of human primordial germ cells (PGCs)^16^. In recent studies, SOX17 has been widely studied in cancers, such as breast cancer^17^, colorectal cancer^18^, hepatocellular carcinoma^19^, gastric cancer^20^, esophageal cancer^21^, cholangiocarcinoma^22^, endometrial cancer^23^ and cervical cancer^24^. However, the majority of these studies are mainly focused on the epigenetic alterations, implying that promoter hypermethylation of SOX17 may contribute to aberrant activation of Wnt/β-catenin signaling pathway^17–19,24–27^. As a transcription factor, the regulatory function of SOX17 on target genes at the transcriptional level contributing to tumorigenesis is insufficiently understood. Furthermore, the molecular mechanisms of SOX17 in cervical carcinoma initiation and progression are largely unknown.

The present study demonstrated that SOX17 was down-regulated during the progression of cervical cancer and that SOX17 expression inhibited the proliferation, tumor formation and activity of the Wnt/β-catenin signaling pathway by directly binding to the promoter region of β-catenin in cervical cancer cells.

## Materials and methods

### Cell lines and human tissue specimens

Five human cervical carcinoma cell lines (HeLa, SiHa, C-33A, CaSki, and HT-3) and SW480 (human colon cancer cell line) were purchased from the American Type Culture Collection (ATCC, Rockville, MD, USA). HeLa, SiHa and C-33A cells were cultured in high-glucose Dulbecco Modified Eagle Medium (DMEM, Sigma-Aldrich, St Louis, MO, USA). CaSki and SW480 cells were cultured in RPMI1640 Medium (Sigma-Aldrich, St Louis, MO, USA). HT-3 cells were cultured in McCoy’s 5A Medium (Sigma-Aldrich, St Louis, MO, USA). All the cell lines were cultured at 37 °C in 5% CO_2_ in the specified media supplemented with 10% fetal bovine serum (FBS, Invitrogen, Carlsbad, CA, USA) and 1% penicillin/streptomycin.

Surgical resection of 67 tumor samples from primary cervical cancer (CC) patients, 20 high-grade squamous intraepithelial lesion (HSIL) and 31 normal cervix (NC) samples obtained from the First Affiliated Hospital of Xi’an Jiaotong University between January 2008 and December 2016 were chosen for immunohistochemistry (IHC). The histology of all CC tissue samples was verified by surgical pathologists. The histological subtype and stage of the tumors were categorized according to the International Federation of Gynecology and Obstetrics (FIGO) classification. Eight normal cervix fresh tissues and eight cervical cancer fresh tissues were collected from the First Affiliated Hospital of Xi’an Jiaotong University for Western blot analysis.

### Immunohistochemistry and immunocytochemistry

Immunostaining of formalin-fixed and paraffin‐embedded tissue was performed on 4 μm paraffin sections using antigen retrieval for 2 min in boiling 10 mM citrate buffer (pH 6.0). Cultured cells were seeded onto cover slips for 48 h and fixed in 4% paraformaldehyde (pH 7.4) at room temperature (RT) for 20 min. After washing three times in PBS, cells were permeabilized with 0.1% Triton X-100. Subsequently, cells or sections were exposed to the blocking solution (PBS/3% hydrogen peroxide) and incubated with primary antibodies overnight at 4 °C. After three washes in PBS, cells or sections were incubated with secondary HRP‐conjugated antibodies for 30 min at RT and counterstained with hematoxylin. As a negative control, PBS was used to replace the primary antibody. For immunostaining analysis, primary goat polyclonal anti-SOX17 (1:50 dilution; #17355, Santa Cruz, CA, USA) and Ki67 (1:100 dilution; #23900, Santa Cruz, CA, USA) were used.

A positive reaction was defined as the observation of a reddish-brown precipitate in the nucleus. The immunoreactivity of immuno-positive sample was determined by multiplying the intensity of the staining (+0 no staining or faint, +1 moderate, +2 strong, and +3 very strong) and percentage of stained area (+0 <5%, +1 5–25%, +2 25–50%, +3 50–75% and +4 >75%)^28^. An overall score of 0 was defined as negative. A score of 1–4 was defined as weak positive, and a score of ≥4 was defined as strong positive. The examination and scoring of all tissues were performed by two investigators independently.

### Western blotting

Cultured cells were lysed in RIPA buffer (50 mM Tris, pH 7.4; 150 mM NaCl; 1% Nonidet P-40; 0.5% sodium deoxycholate; and 0.1% sodium dodecyl sulfate), which was supplemented with a protease inhibitor. After incubation, lysates were cleared by centrifugation and then subjected to protein concentration assay (BCA kit, Thermo Scientific, New York, NY). Tissue samples were homogenized in RIPA buffer supplemented with protease inhibitors using a blender. Lysates were subjected to SDS-PAGE, transferred to PVDF membranes, blocked with 5% non-fat milk and then incubated with primary antibodies overnight at 4 °C. Immunoreactive bands were incubated with HRP‐conjugated secondary antibodies (Thermo Fisher Scientific, New York, NY, USA) for 1 h at RT and visualized with an enhanced chemiluminescence reagent (Millipore, Billerica, MA, USA). The membrane was then scanned with a protein-imprinting imaging system (Tanon 5200, China). The SOX17 Western blot results were normalized to those of GAPDH for quantification.

Primary antibodies used were anti-SOX17 (1:500 dilution; #17355, Santa Cruz, CA, USA), anti-GAPDH (1:1000 dilution; #47724, Santa Cruz, CA, USA), p-GSKβ (1:1000 dilution; #11757, Santa Cruz, CA, USA), β-catenin (1:1000 dilution; #7963, Santa Cruz, CA, USA), c-Myc (1:1000 dilution, #sc-40, Santa Cruz, CA, USA), and cyclin D1 (1:1000 dilution, #8396, Santa Cruz, CA, USA). Secondary antibodies used were either horseradish peroxidase-conjugated anti-goat or anti-mouse immunoglobulin G (IgG; Thermo Fisher Scientific, New York, NY).

### Cell growth and cell viability assays

Cells (2 × 10^4^) were seeded in 6-well plates in triplicate and counted every 2 days for 1 week using a hemocytometer. Cell growth curves were generated to assess cell proliferation. For cell viability assays, cells were plated at a density of 2,000 cells per well and evaluated by 3-(4,5-dimethylthiazoleyl)-2,5-diphenyl tetrazolium bromide (Sigma-Aldrich, St Louis, MO, USA) dye for 7 days according to standard protocol. The number of viable cells was detected by measuring the absorbance at 490 nm. The experiment was repeated in three independent experiments.

### Tumor xenograft assay

All animal experiments were conducted in accordance with the animal use guidelines from the Animal Care and Use Committee of the Medical School of Xi’an Jiaotong University. 6–7-week-old female BALB/c-nude mice (Slac Laboratory Animal Co., Ltd., Shanghai, China) were housed in a specific pathogen free (SPF) room with constant temperature (22–25 °C) and humidity (40–50%). Cervical carcinoma cells (1 × 10^6^) in the exponential growth phase were harvested and injected (100 μL per site) into the subcutis on the dorsum of each mouse. Tumors were measured in two dimensions by using manual calipers. Tumor volume was calculated using the following formula: *V* = 0.5 × length × width × width. Tumor volume was measured every 2–3 days.

### Flow cytometry

Cells (1 × 10^6^) were harvested by trypsinization and fixed by 70% ethanol (−20 °C). Cells were then stained with solutions containing propidium iodide (50 μg/ml, Sigma Aldrich, St. Louis, MO, USA) and DNase-free RNase A (50 μg/ml, Sigma Aldrich, St. Louis, MO, USA). After 30 min of incubation, samples were washed and re-suspended in PBS. The samples were run on a FACS Calibur flow cytometer (BD Biosciences, San Jose, CA, USA) using CellQuest software. All data were analyzed with FlowJo_V10 cytometer software.

### Genomic DNA extraction and sodium bisulfite modification

Genomic DNA was isolated from cells using the MiniBEST Universal Genomic DNA Extraction Kit (Takara, Osaka, Japan) and stored at −20 °C before use. DNA was modified with EpiTect® Bisulfite Kit (Qiagen, Hilden, Germany) according to the instructions. Briefly, 0.5 μg denatured DNA was treated with sodium bisulfite at 95 °C for 5 min, 60 °C for 25 min, 95  °C for 5 min, 60 °C for 85 min, 95 °C for 5 min and then 60 °C for 175 min. Samples were then applied to supplied columns, subjected to column clean up.

### RNA extraction, real-time (RT) qPCR and methylation specific PCR (MSP)

Total RNA was isolated from cell lines by TRIzol reagent (Invitrogen, Carlsbad, CA, USA), and cDNA was synthesized using PrimeScript RT reagent Kit (Takara, Osaka, Japan). Total cDNA was used as a template for PCR amplification with GAPDH as an internal control. Real-time quantitative PCR was performed in triplicate for each primer set and each cell sample using an Mx3000P QPCR System (Agilent Technologies, Santa Clara, CA) and the SYBR Premix ExTaq II (Takara, Osaka, Japan). Results were analyzed via the ∆∆Ct method using GAPDH as the housekeeping gene. The specific primers are listed in Table S2.

TaKaRa EpiTaq™ HS DNA polymerase was used for methylation specific PCR. Thermocycling conditions used were one cycle at 94 for 5 min; 40 cycles at 98 for 10 s, 61 for 30 s, and 72 for 30 s; and final extension at 72 for 5 min. PCR products were loaded with 2% agarose gels followed by staining with ethidium bromide and directly visualized under UV illumination. SW480 methylated DNA and ultra-pure water were used as positive^18^ and negative controls, respectively. The specific primers are selected from a previous reference^29^ and listed in Table S2.

### Plasmids and cell transfection

Full-length SOX17 and β-catenin cDNA was amplified (primers seen in Table S2). SOX17 and β-catenin cDNA was subcloned into pIRES2-AcGFP (Clontech, Mountain View, CA) to generate pIRES2-AcGFP-SOX17 and pIRES2-AcGFP-CTNNB1 expressing plasmids.

The small interfering RNA expression vector that expresses a SOX17-specific short hairpin RNA (shRNA) with the vector bone of pGPU6/GFP/Neo was purchased from GenePharma Co., Ltd (C02007, genepharma, Shanghai, China). The primers were described in Table S2. Lipofectamine 2000 reagent (Invitrogen, Carlsbad, CA, USA) was used for transfection according to the manufacturer’s instructions. After treatment with G418 (Calbiochem, La Jolla, CA, USA) for 3 weeks, the transfected cells were collected and expanded, and then the drug-resistant colonies were identified.

### Microarrays and gene expression analysis

Three HeLa-GFP cells and three HeLa-SOX17 cells were used for microarrays. Total RNA was extracted using TRIzol reagent (Invitrogen, Carlsbad, CA, USA). An Affymetrix PrimeView Human Gene Expression Array was used to investigate the changes in transcriptional profiles. The experiment was performed based on the manufacturer’s standard protocols. Genes with ≥1.5-fold change between two groups were identified as differentially expressed genes.

### Luciferase reporter assay

Fragments of the β-catenin promoter (predicted from position −2000 bp to +44 bp), cyclin D1 promoter (predicted from position −2024 bp to +200 bp) and c-Myc promoter (predicted from position −1975 bp to +249 bp) were cloned into the pGL3-Basic Vector (Promega, Madison, WI, USA) to generate β-catenin, cyclin D1 and c-Myc promoter reporter constructs, respectively. All constructs were verified by sequencing. Cells were seeded in 24-well plates and transiently transfected with plasmids containing firefly luciferase reporters and recombinant promoter reporter constructs. The luciferase activity was measured after incubation for 48 h using the Dual Luciferase Assay kit (Promega, Madison, WI). All experiments were performed as three independent experiments. The transfection efficiency was normalized with Renilla luciferase activity. The specific promoter activity was presented as the change in the experimental group vs. the control group. The primers and oligonucleotides are listed in Table S2. The specific activity is shown as the fold change of the experimental group vs. the control group.

### Quantitative chromatin immunoprecipitation

Cervical carcinoma cells were subjected to ChIP using the EZ-Magna ChIP Assay kit (Millipore, Darmstadt, Germany). Cells were fixed in 1% formaldehyde solution and incubated at room temperature for 10 min followed by the addition of 2 mL of 10X glycine to each dish to quench the unused formaldehyde. Cells were washed twice with PBS and scraped into a microcentrifuge tube, and cells were then centrifuged at 800x*g* at 4°C to pellet cells. Cell pellets were sonicated to shear the chromatin to a manageable size. After sonication, chromatin–protein complexes were immunoprecipitated with 5 μg of anti-SOX17 antibodies (#17355, Santa Cruz, CA, USA) and 20 μL of fully resuspended protein A/G magnetic beads. For the negative control, 1 μg of normal mouse IgG was used. Beads were then washed two times with sonication buffer, and DNA was eluted in elution buffer. Cross-links were reversed overnight. RNA and protein were digested using RNase and Proteinase K, respectively, and DNA was purified with phenol chloroform extraction and ethanol precipitation. Real-time PCR was performed to amplify the regions of interest or internal negative control regions. Each sample was assayed in triplicate, and the fold enrichment ratio was calculated as the value of the ChIP sample vs. the corresponding input sample. Samples that yielded a two-fold enrichment or better were considered positive targets. The primers used for these studies are listed in Table S2.

### Oncomine^TM^ database

Meta-analysis of SOX17 in multiple cancers was performed using The Oncomine^TM^ database (Compendia Bioscience, AnnArbor, MI, USA, http://Oncomine.org). Four datasets^30–33^ were identified using the search parameters “Gene: SOX17”, “Cancer Type: Cervical Cancer”, “Analysis Type: Cancer vs. Normal Analysis” and “Data Type: mRNA”. Then, the search parameters “Gene: SOX17”, “Analysis Type: Cancer vs. Normal Analysis”, “Data Type: mRNA” and “Dataset Size: 151+ samples” were used and the following studies were included: Lee Bladder^34^, The Cancer Genome Atlas (TCGA) Brain, TCGA Breast, TCGA Colorectal, Cui Gastric^35^, Chen Liver^36^, Hou Lung^37^, Agnelli Myeloma^38^, TCGA Ovarian, and Barretina Sarcoma^39^. A summary of SOX17 expression in multiple cancer types was generated.

### Statistical analysis

Statistical analysis was performed with SPSS 19.0 software (SPSS Inc., Chicago, IL). All data are expressed as the group means ± standard deviation of the mean (SD). The two-tailed Chi-square test or Fisher’s exact test was used to determine the significance of the differences between the covariates. A univariate analysis was analyzed by Student’s *t*-test (two-tailed) and the Mann–Whitney U-test. The expression variance analysis of CESC was based on the Wilcox test. Meta-analyses were performed as described^40^. A *p* value of <0.05 was considered statistically significant. For comparison among groups, the Chi-square test or one-way ANOVA was performed.

## Results

### Expression of SOX17 in normal human cervix and various cancerous cervical lesions

**Fig. 1:**
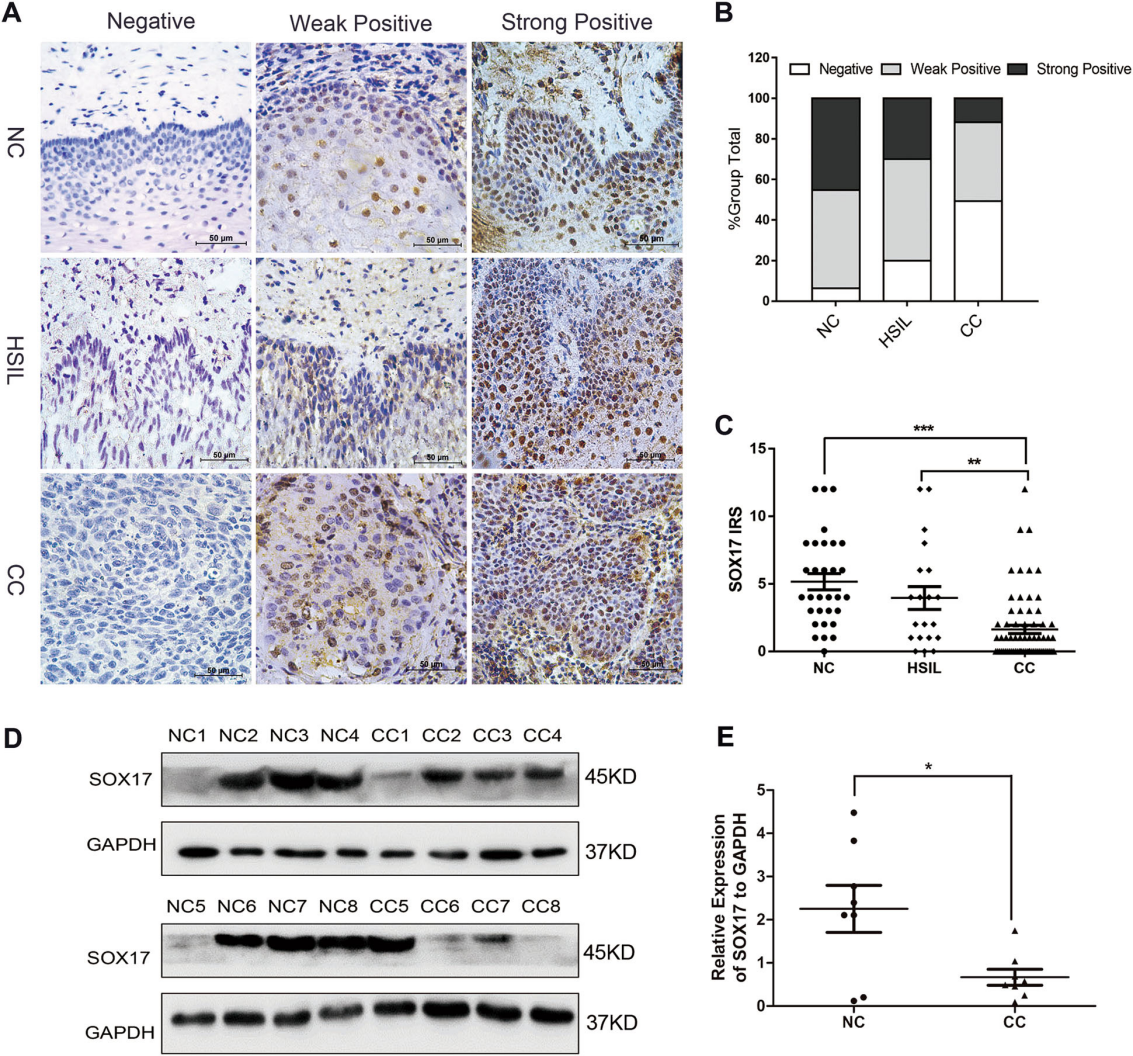
The expression of SOX17 in the normal human cervix and various cancerous cervical lesions. **a** Immunohistochemical detection of SOX17 in normal cervix, high-grade squamous intraepithelial lesion (HSIL) and cervical cancer samples; original magnification, 1000×. **b** SOX17 staining is classified into three categories (negative, weak positive and strong positive), and the bar chart shows the percentage of each group (31 normal cervix specimens, 20 HSIL specimens, and 67 cervical cancer tissue specimens). **c** The scatter plot shows the immunoreactivity scores obtained for the staining of SOX17 in normal cervix, HSIL and invasive cervical cancer samples (points represent the IHC score per specimen, and one-way ANOVA was performed). **d** The expression of SOX17 in normal cervix (NC) and cervical carcinoma (CC) samples was detected using western blotting. **e** The relative expression of SOX17 in each normal cervix tissue (*n* = 8) and cervical cancer tissue sample (*n* = 8) is shown. The data shown are the ratios of the SOX17/GAPDH of each specimen and the means ± standard error of the NC and CC groups. *p *<* 0.05, *p<0.01, ***p *<* 0.001

The expression status of SOX17 was first evaluated in normal human cervix (NC), high-grade squamous intraepithelial lesion (HSIL) and cervical cancer (CC) samples by immunohistochemistry (IHC). Representative SOX17 staining was observed in the nucleus and/or cytoplasm of positive cells in various cervical tissues (Fig. 1a). It showed that 49.3% (33/67) of CC demonstrated reduced SOX17 expression compared to NC (6.4%, 2/31) and HSIL (20%, 4/20) (Fig. 1b). Significant differences were observed between HSIL and CC samples as well as CC and NC samples, but not observed between NC and HSIL samples (Table S1, NC vs. HSIL, *p* > 0.05; NC vs. CC, *p* < 0.05; HSIL vs. CC, *p* < 0.05). Moreover, immunoreactivity score (IRS) of SOX17 staining revealed that SOX17 expression was significantly decreased from NC (5.16 ± 3.34) to HSIL (3.95 ± 3.79) and finally to CC (1.63 ± 2.50) (Fig. 1c, NC vs. HSIL, *p* *>* 0.05; NC vs. CC, *p* < 0.001; HSIL vs. CC, *p* < 0.05). To further confirm the SOX17 expression results in cervical carcinogenesis, western blot analysis was performed to detect the SOX17 expression in eight randomly selected NC and eight CC fresh specimens (Fig. 1d). The average SOX17 expression level was lower in cervical carcinoma tissues than in normal cervix tissues (Fig. 1e, 0.67 ± 0.52 vs. 2.25 ± 1.54, *p* < 0.05). All the results indicated that SOX17 expression is negatively related to cervical carcinogenesis.

### SOX17 suppresses tumor formation of cervical cancer cells in vivo

**Fig. 2:**
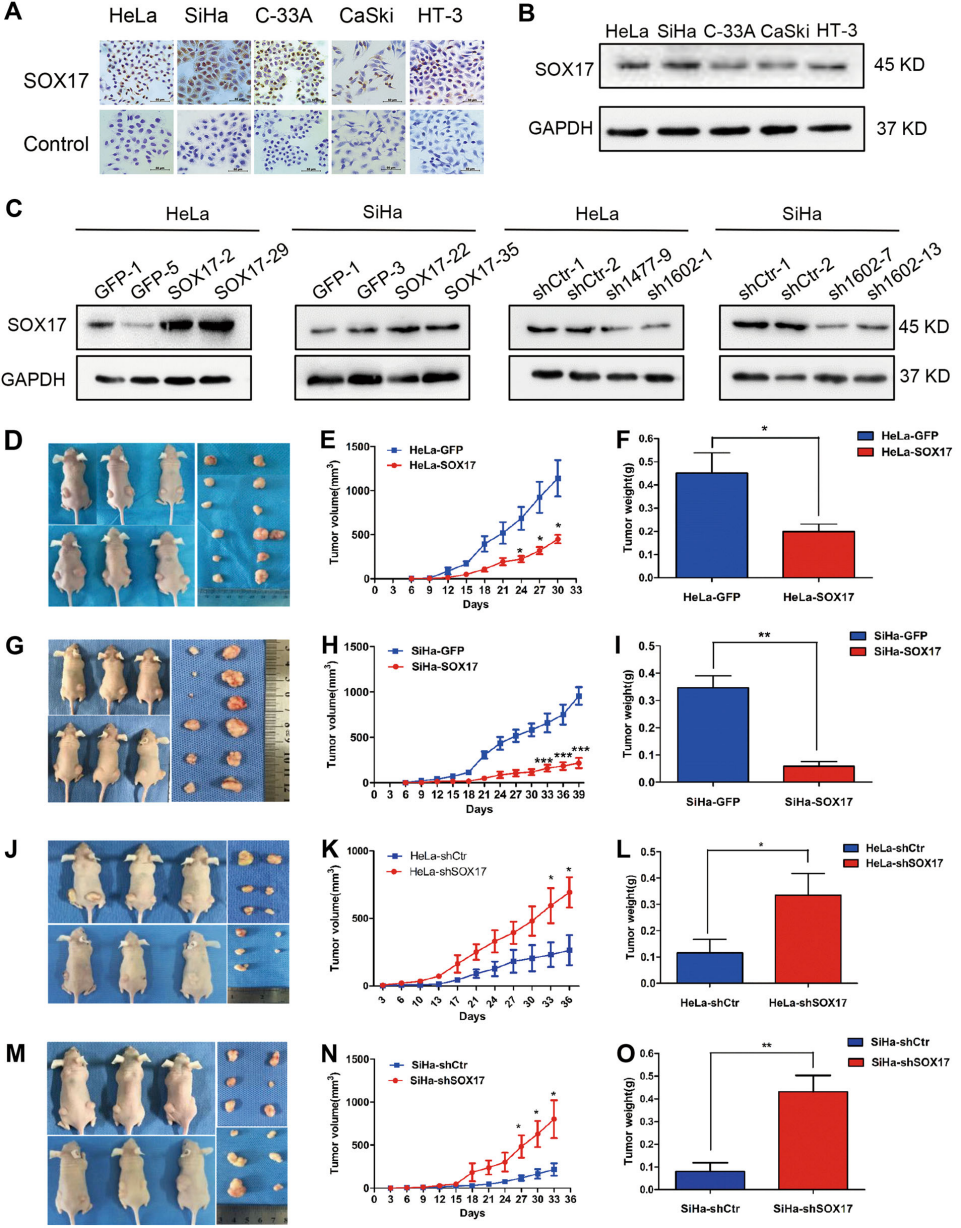
SOX17 suppressed cervical carcinoma tumor growth in vivo. SOX17 expression in human cervical cancer cell lines was detected using immunocytochemistry (**a**) and Western blotting (**b**). Stably transfected cell lines were identified by western blotting. **c** HeLa-GFP and HeLa-SOX17 cells; SiHa-GFP and SiHa-SOX17 cells; HeLa-shControl and HeLa-shSOX17 cells; SiHa-shControl and SiHa- shSOX17 cells. Female nude mice were injected with the SOX17-modified cervical cancer cells (left) and control cells (right) (**d**, **g**, **j**, **m**). Tumor growth curves were calculated after injection based on monitoring performed every 3 days and the xenograft tumors were dissociated and weighed at the end of the experiment. **e**, **f** HeLa-GFP and HeLa-SOX17 cells. **h**, **i** SiHa-GFP and SiHa-SOX17 cells. **k**, **l** HeLa-shControl and HeLa-shSOX17 cells. **n**, **o** SiHa-shControl and SiHa-shSOX17 cells. Values are shown as the mean ± SD. *p *<* 0.05, **p *<* 0.01, ***p *<* 0.001

Immunocytochemical assays, RT-PCR and western blot analyses were performed in cervical cancer cell lines. It is revealed that SOX17 expression was found in cervical cancer cell lines (Fig. 2a, b and S1A). The MSP was performed and showed different levels of methylation of SOX17 in cervical cancer cells. To assess the effects of  SOX17 expression in tumor formation, stable SOX17 overexpression and stable SOX17 knockdown cell lines were established. Western blot analysis showed that the SOX17 expression was effectively up-regulated and down-regulated, respectively. (Fig. 2c and Figure S1C).

To identify the effect of SOX17 on tumor formation of cervical cancer cells in vivo, SOX17-modified cells (left side) and control cells (right side) were subcutaneously inoculated into each posterior flank of the same female nude mouse at the same time. The palpable tumors formed by HeLa cells grew more slowly, smaller (Fig. 2d, e, *p* < 0.05) and lighter (Fig. 2f, 0.20 ± 0.08 g vs. 0.45 ± 0.21 g, *p* < 0.05) after SOX17 overexpression. Similar results were found in the SiHa-SOX17 cells (Fig. 2g, h, *p* < 0.001; Fig. 2i, 0.06 ± 0,04 vs. 0.35 ± 0.11, *p* < 0.01). Conversely, the tumors grew faster, larger (Fig. 2j, k, *p* < 0.05) and heavier (Figs. 2l, 0.33 ± 0.20 vs. 0.12 ± 0.12 *p* < 0.05) after SOX17 knockdown in HeLa cells. Similar results were found in the SiHa-shSOX17 cells (Fig. 2m, n, *p* < 0.05; Fig. 2o, 0.43 ± 0.18 vs. 0.08 ± 0.09, *p* < 0.01). All these results demonstrated that the SOX17 protein suppresses tumor formation of cervical cancer cells in vivo.

### SOX17 inhibits tumor formation by suppressing the proliferation of cervical cancer cells in vivo and in vitro

**Fig. 3:**
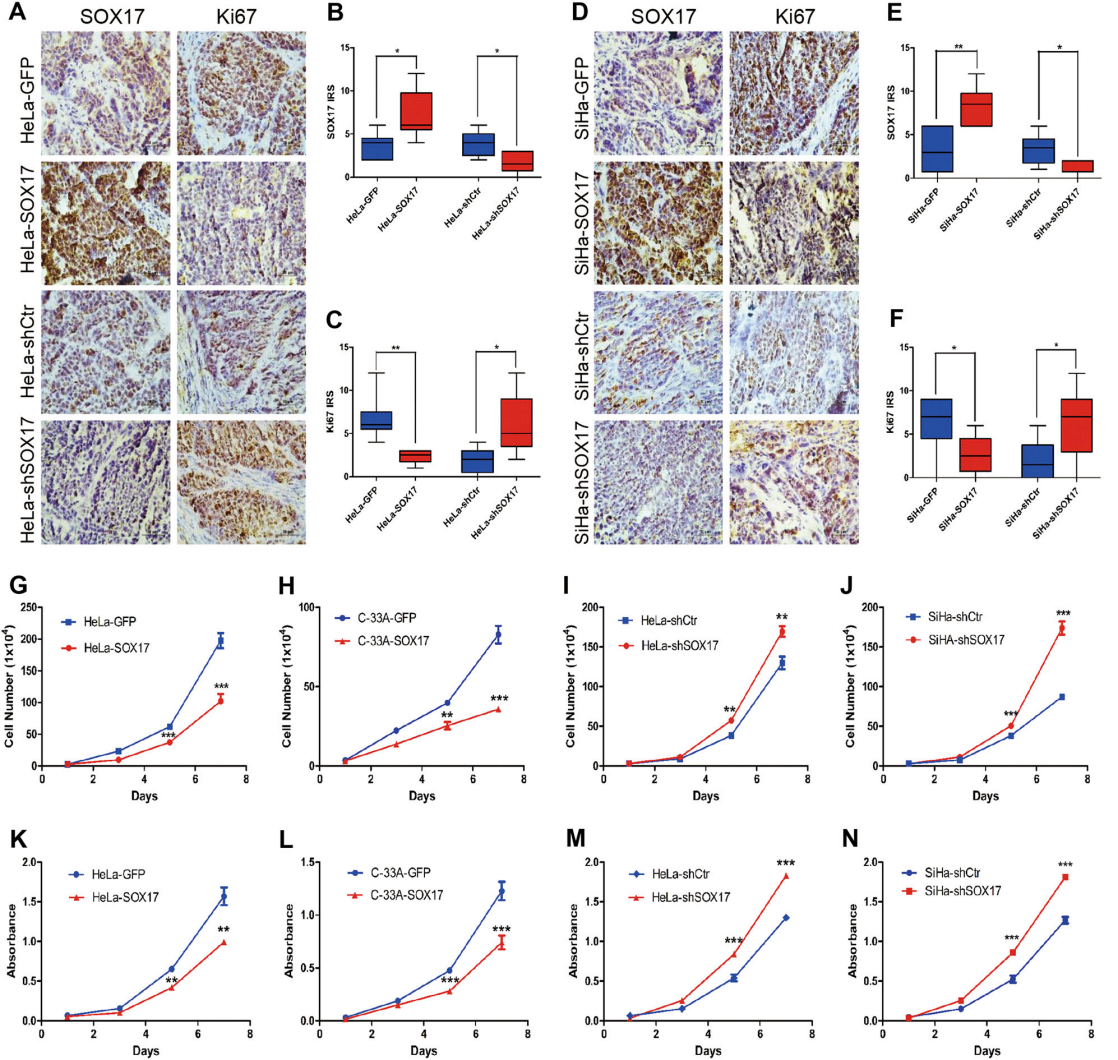
SOX17 inhibits tumor formation by suppressing the proliferation of cervical cancer cells in vivo and in vitro. Immunohistochemical staining (**a**) and immunoreactivity scores of SOX17 (**b**) and Ki-67 (**c**) in xenograft tumor tissues derived from HeLa-GFP cells, HeLa-SOX17 cells, HeLa-shcontrol cells and HeLa-shSOX17 cells. Immunohistochemical staining (**d**) and immunoreactivity scores of SOX17 (**e**) and Ki-67 (**f**) in xenograft tumor tissues derived from SiHa-GFP cells, SiHa -SOX17 cells, SiHa -shcontrol cells and SiHa -shSOX17 cells. The proliferation was detected using growth curves in HeLa-GFP and HeLa-SOX17 cells (**g**), C33A-GFP and C33A-SOX17 cells (**h**), HeLa-shControl and HeLa- shSOX17 cells(**i**) and SiHa-shControl and SiHa- shSOX17 cells (**j**). The viability was detected by the MTT assay in HeLa-GFP and HeLa-SOX17 cells (**k**), C33A-GFP and C33A-SOX17 cells (**l**), HeLa-shControl and HeLa-shSOX17 cells (**m**) and SiHa-shControl and SiHa- shSOX17 cells (**n**). Data were statistically analyzed with student’s *t*-test and values are shown as the mean ± SD. * *p* *<* 0.05, ** *p* *<* 0.01, *** *p* *<* 0.001

To explore if  SOX17 suppresses the tumor formation of cervical cancer cells by inhibiting cell proliferation, the expression of Ki67 was evaluated in the tumor xenograft tissues by IHC. After SOX17 overexpression in HeLa cells, the tumors displayed a weaker Ki67 staining and lower IRS (Fig. 3a-c; 3.67 ± 1.51 vs. 7.17 ± 2.86, *p* < 0.05, 3.80 ± 1.48 vs. 1.67 ± 1.21, *p* < 0.05). In contrast, SOX17 knockdown induced the Ki67 staining and IRS of tumor tissues weaker and lower, respectively, (Fig. 3a–c; 6.67 ± 2.73 vs. 2.33 ± 0.82, *p* < 0.01, 1.80 ± 1.48 vs. 6.00 ± 3.58, *p* < 0.05). Similar results were found SiHa SOX17-overexpressing and knockdown cells (Fig. 3d–f; 3.17 ± 2.56 vs. 8.33 ± 2.25, *p* < 0.01, 3.33 ± 1.75 vs. 1.50 ± 0.84, *p* < 0.05; 6.33 ± 3.39 vs. 2.67 ± 2.16, *p* < 0.05, 2.00 ± 2.28 vs. 6.33 ± 4.08, *p* < 0.05). All these results indicated that SOX17 suppresses the tumor formation of cervical cancer cells in vivo by inhibiting the cell proliferative ability.

To verify if SOX17 inhibits proliferation of cervical cancer cells in vitro, cell growth curve assays and MTT assays were performed in vitro. As shown in Fig. 3a, b, up-regulating SOX17 resulted in a significant growth inhibition of HeLa, C-33A and SiHa cells (Fig. 3g, *p* < 0.001; Fig. 3h, *p* < 0.001; Figure S1D, p < 0.001). Conversely, SOX17 knockdown markedly promoted cell growth (Fig. 3i, *p* < 0.01; Fig. 3j, *p* < 0.001). In addition, MTT assays showed that SOX17 overexpression resulted in a significant decrease of cell viability in HeLa, C-33A and SiHa cells (Fig. 3k, *p* < 0.01; Fig. 3l, *p* < 0.001; Figure S1D, *p* < 0.001), and knockdown of SOX17 led to a marked increase of cell viability in HeLa and SiHa cells (Fig. 3m, *p* < 0.001; Fig. 3n, *p* < 0.001). These data suggested that SOX17 also suppresses the proliferation and viability of cervical cancer cells in vitro. Collectively, all these results indicated that SOX17 suppressed the tumor formation of cervical cancer cells by inhibiting the cell proliferative ability in vivo and in vitro.

### SOX17 inhibits the proliferation of cervical carcinoma cells through arresting at the cell transition from G0/G1 phase to S phase

**Fig. 4:**
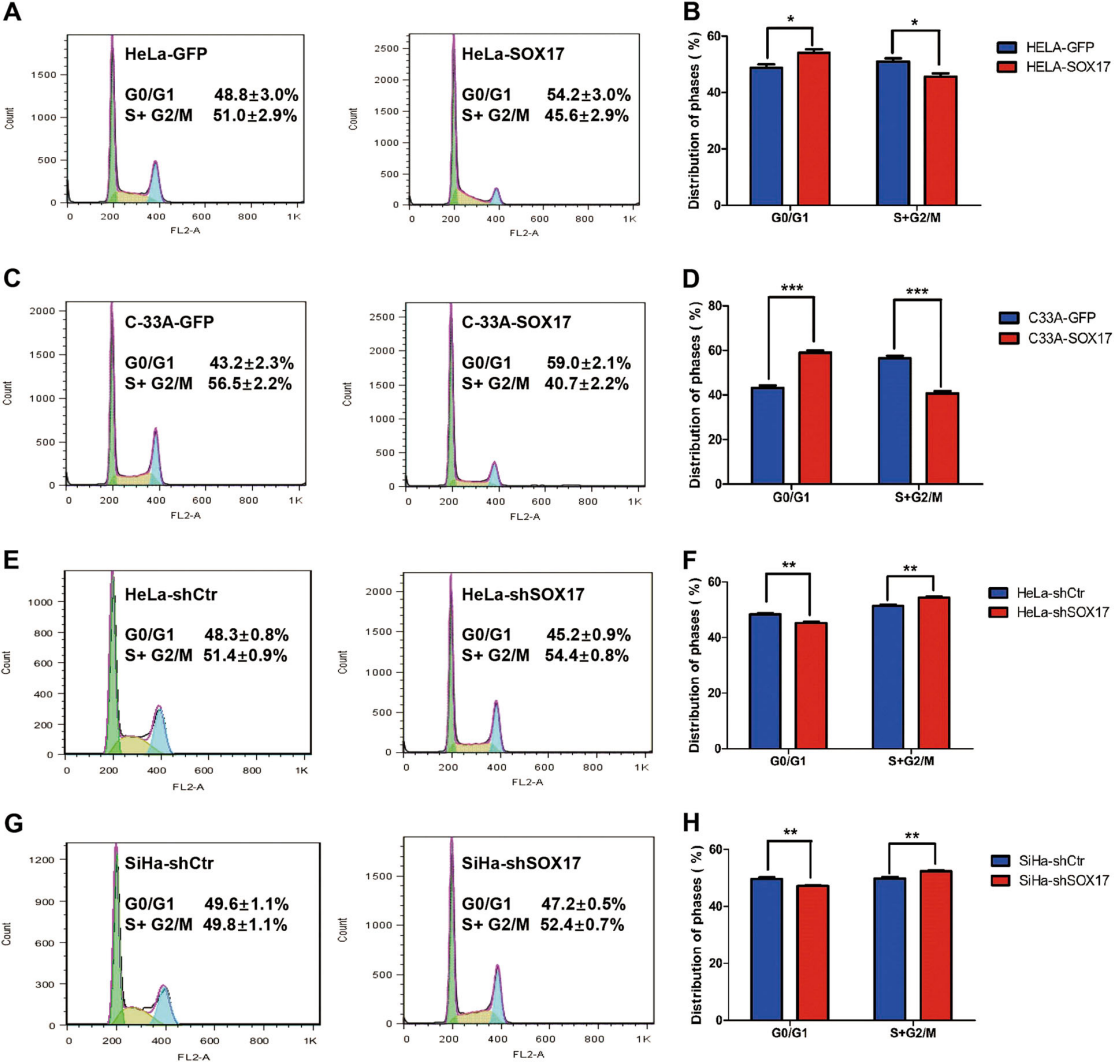
SOX17 inhibits the proliferation of cervical carcinoma cells through arresting at the cell transition from G0/G1 phase to S phase. In the flow cytometry figures, the y axis shows the count of effective cells and the x axis shows the DNA content. Each colored area represents the cells of different phases of cell cycle: green area refers to the cells in the G_0_/G_1_ phase, yellow area refers to the cells in the S phase and blue area refers to the cells in the G_2_/M phase. The cell cycles of HeLa-GFP and HeLa-SOX17 cells were analyzed using flow cytometry (**a**), and a quantitative analysis of the cell cycle are shown (**b**). The cell cycles of C33A-GFP and C33A-SOX17 cells (**c**) and the quantitative analysis (**d**) are shown . The cell cycles of HeLa-shControl and HeLa-shSOX17 cells (**e**) and the quantitative analysis (**f**) are shown. The cell cycles of SiHa-shControl and SiHa-shSOX17 cells (**g**) and the quantitative analysis (**h**) are shown. The data were shown as the mean ± SD of three independent experiments. *p *<* 0.05, **p *<* 0.01, ***p *<* 0.001 vs. control using One-Way ANOVA

To further investigate the mechanism by which SOX17 inhibits the proliferation of cervical cancer cells, FACS was performed for cell cycle analysis on SOX17-modified cervical cancer cells and control cells. As shown in Fig. 4a, b, SOX17 overexpression caused a significant increase of G_0_/G_1_ phase (54.2 ± 3.0 vs. 48.8 ± 3.0%, *p* < 0.05) and a great decrease of S/G_2_/M phase (45.6 ± 2.9 vs. 51.0 ± 2.9%, *p* < 0.05) in the percentage of HeLa cells. Similar results were observed in C-33A (Fig. 4c, d, 59.0 ± 2.1 vs. 43.2 ± 2.3%, *p* < 0.001; 40.7 ± 2.2 vs. 56.5 ± 2.2%, *p* < 0.001) and SiHa cells (Figure S1F, 53.78 ± 0.46 vs. 47.45 ± 1.01%, *p* < 0.001; 48.8 ± 3.0 vs. 54.2 ± 3.0%, *p* < 0.001). Conversely, SOX17 knockdown resulted in a marked decrease in the percentage of HeLa cells (Fig. 3e, f, 48.3 ± 0.8 vs. 45.2 ± 0.9%, *p* < 0.01) and SiHa cells (Fig. 3g, h, 49.6 ± 1.1 vs. 47.2 ± 0.5%, *p* < 0.01) in G_0_/G_1_ phase as well as an increase in the percentage of cells in S/G_2_/M phase (51.4 ± 0.9 vs. 54.4 ± 0.8%, *p* < 0.01; 49.8 ± 1.1 vs. 52.4 ± 0.7%, *p* < 0.01). Collectively, these results indicated that SOX17 suppressed the proliferation of cervical cancer cells through arrest at the cell transition from G_0_/G_1_ phase to the S phase.

### SOX17 induces cell cycle arrest by trans-suppressing the Wnt/β-catenin pathway in cervical cancer cells

**Fig. 5:**
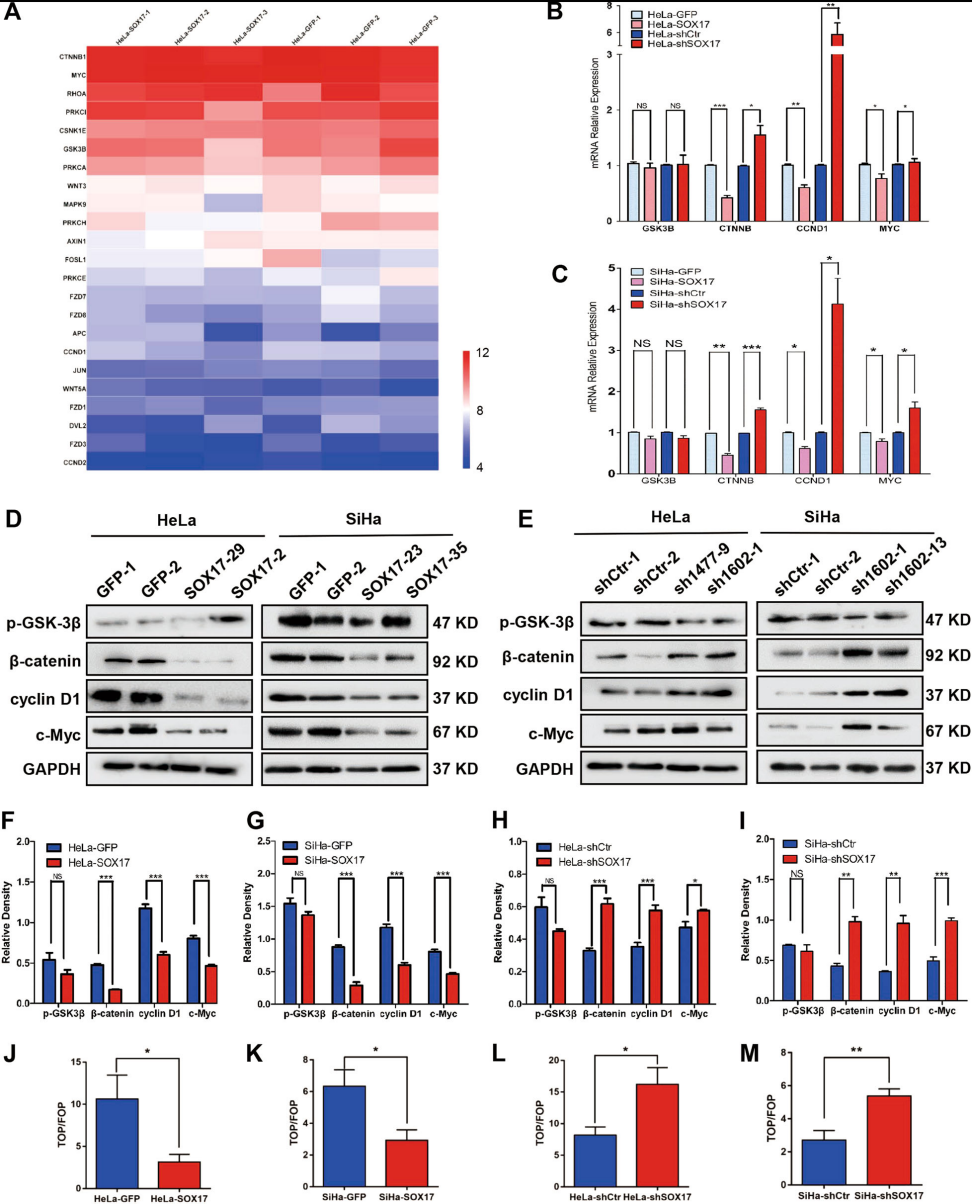
SOX17 induces cell cycle arrest by trans-suppressing the Wnt/β-catenin pathway in cervical cancer cells. **a** Visualization of known genes for Wnt/β-catenin pathway in HeLa-GFP cells (right) and HeLa-SOX17 cells (left) as a heatmap using microarray. Data were log2 normalized. Real-time PCR analysis is shown for the mRNA levels of Wnt/β-catenin pathway key genes in SOX17 overexpression and knockdown HeLa (**b**) and SiHa (**c**) cells. The expression of Wnt/β-catenin pathway key proteins in SOX17 modified HeLa and SiHa cells were determined by western blot (**d**, **e**) and the quantitative analysis (**f**–**i**) was shown. **j**–**m** SOX17 modified HeLa and SiHa cells were transfected with the TOP/FOP-Flash reporter plasmid, and the reporter activities were detected 48 h after transfection by a luciferase assay. Data represent mean ± SD of triplicate experiments and statistical analysis was done with Student’s *t*-test. * *p* *<* 0.05, ** *p* *<* 0.01, *** *p* *<* 0.001

To explore the molecular mechanism by which SOX17 inhibits cervical carcinogenesis, we investigated the mRNA expression levels in three HeLa-SOX17 cell lines and three HeLa-GFP cell lines by microarray analyses. We detected a total of 49,293 transcripts, and identified that 28,123 were up-regulated and 21,170 were down-regulated. Of these transcripts, we identified that the expression of 23 Wnt/β-catenin signaling pathway-related genes was changed after up-regulating SOX17 (Fig. 5a). To further confirm the microarray results, we conducted real-time PCR to measure mRNA levels of the key molecules of the Wnt/β-catenin signaling pathway, including GSK3B, CTNNB, CCND1 and MYC^52–54^. It showed that the mRNA levels of CTNNB, CCND1, and MYC (normalized by GAPDH) were significantly decreased in SOX17 overexpression cells and increased in SOX17 knockdown cells (Fig. 5b, c, S2A). However, the mRNA expression of GSK3B failed to show a significant fold change (Fig. 5b, c, S2A, *p* *>* 0.05). These phenomena were also confirmed in the SOX17-modified SW480 cell lines (Figure S2B). All the data above suggested that SOX17 trans-suppressed the expression of the Wnt/β-catenin signaling pathway-related genes CTNNB, CCND1 and MYC, but not GSK3B, at the transcriptional level.

Western blot analysis was used to further detect the expression of key molecules of the Wnt/β-catenin signaling pathway, including p-GSK3β, β-catenin, cyclin D1 and c-Myc, at the protein level. Consistent with the mRNA results, a significant decrease of β-catenin, cyclin D1, and c-Myc protein expression was found in the SOX17 overexpressing cervical cancer cells (Fig. 4d, f, g, Figs. S2C, D). These phenomena were also found in the SW480 cell (Figure S2E). In contrast, the expression levels of these proteins in SOX17- knockdown cervical cancer cells were significantly increased (Fig. 4e, h, i). However, the expression of p-GSK3β failed to show significant change (Fig. 4f–i, S1D and S2E). These results demonstrated that SOX17 suppressed the expression of key molecules of the Wnt/β-catenin pathway, including β-catenin, cyclin D1, and c-Myc, but not p-GSK3β, at the protein level.

Although β-catenin, cyclin D1, and c-Myc were markedly inhibited by SOX17, p-GSK3β failed to show significant expression change. These data are insufficient to conclude that SOX17 suppresses the activity of the Wnt/β-catenin signaling pathway. The TOP/FOP Flash luciferase reporter assay was conducted to detect Wnt/β-catenin signaling activity. Compared to control cells, SOX17 overexpression led to a significant inhibition of TOP-Flash reporter activity in HeLa, SiHa and C-33A cells (Fig. 5j, *p* < 0.05; 5 K, *p* < 0.05; S1F, *p* < 0.05), whereas SOX17 knockdown dramatically increased the activity in HeLa and SiHa cells (Fig. 5l, *p* < 0.05; 5 M, *p* < 0.01). Also, these phenomena were observed in SW480 cell (Figure S2G, *p* < 0.05; S2H, *p* < 0.01). All these data suggested that the activity of the Wnt/β-catenin signaling pathway is attenuated by SOX17 in cervical cancer cells.

### Up-regulating β-catenin attenuates the proliferation suppression of SOX17 in cervical cancer cells

All of these three molecules, β-catenin, cyclin D1 and c-Myc, were down-regulated by SOX17 in cervical cancer cells, but β-catenin is the upstream molecule of cyclin D1 and c-Myc in Wnt/β-catenin signaling pathway. Thus, the recombinant β-catenin plasmid was transiently transfected into the SOX17-overexpressing cells to detect if the proliferation suppression of cervical cancer cells by SOX17 could be rescued. After transfection of pIRES2-AcGFP-CTNNB1, β-catenin, cyclin D1 and c-Myc expression levels were elevated in SOX17-overexpressing cells compared to controls at both protein level (Figure S3A-C, *p* > 0.05; *p* > 0.05) and mRNA level (Fig. S3D, E, *p* > 0.05; *p* > 0.05). Simultaneously, SOX17-overexpressing cells transfected with pIRES2-AcGFP-CTNNB1 showed higher rate of growth and viability than cells transfected with empty vectors (Figure S3F-I, *p*>0.05). As shown in Figure S3J,K, β-catenin overexpression significantly decreased the percentage of cervical cancer cells in G_0_/G_1_ phase and increased the percentage in S/G_2_/M phase (HeLa-SOX17-β-catenin vs. HeLa-GFP-Vector, *p* *>* 0.05; C-33A-SOX17-β-catenin vs. C-33A-GFP-Vector, *p* > 0.05). These results suggested that β-catenin might be the key molecule by which SOX17 suppressed the proliferation of cervical cancer cells by inhibiting the activity of the Wnt/β-catenin signaling pathway.

To validate the correlation between the expression of SOX17 and β-catenin in clinical cervical cancer specimens, their expression levels were detected by western blot (Figure S3L). The results showed that as the SOX17 expression increased, β-catenin expression decreased in the human cervical cancer tissues. The logistical regression analysis showed that SOX17 expression was significantly negatively correlated with β-catenin (Figure S3M, *R* = −0.5085, *p* < 0.05). These results supported that SOX17 acted as a negative regulator of β-catenin in clinical cervical cancer tissues.

### SOX17 inhibits the activity of Wnt/β-catenin pathway through directly binding to the promoter of β-catenin in cervical cancer cells

**Fig. 6:**
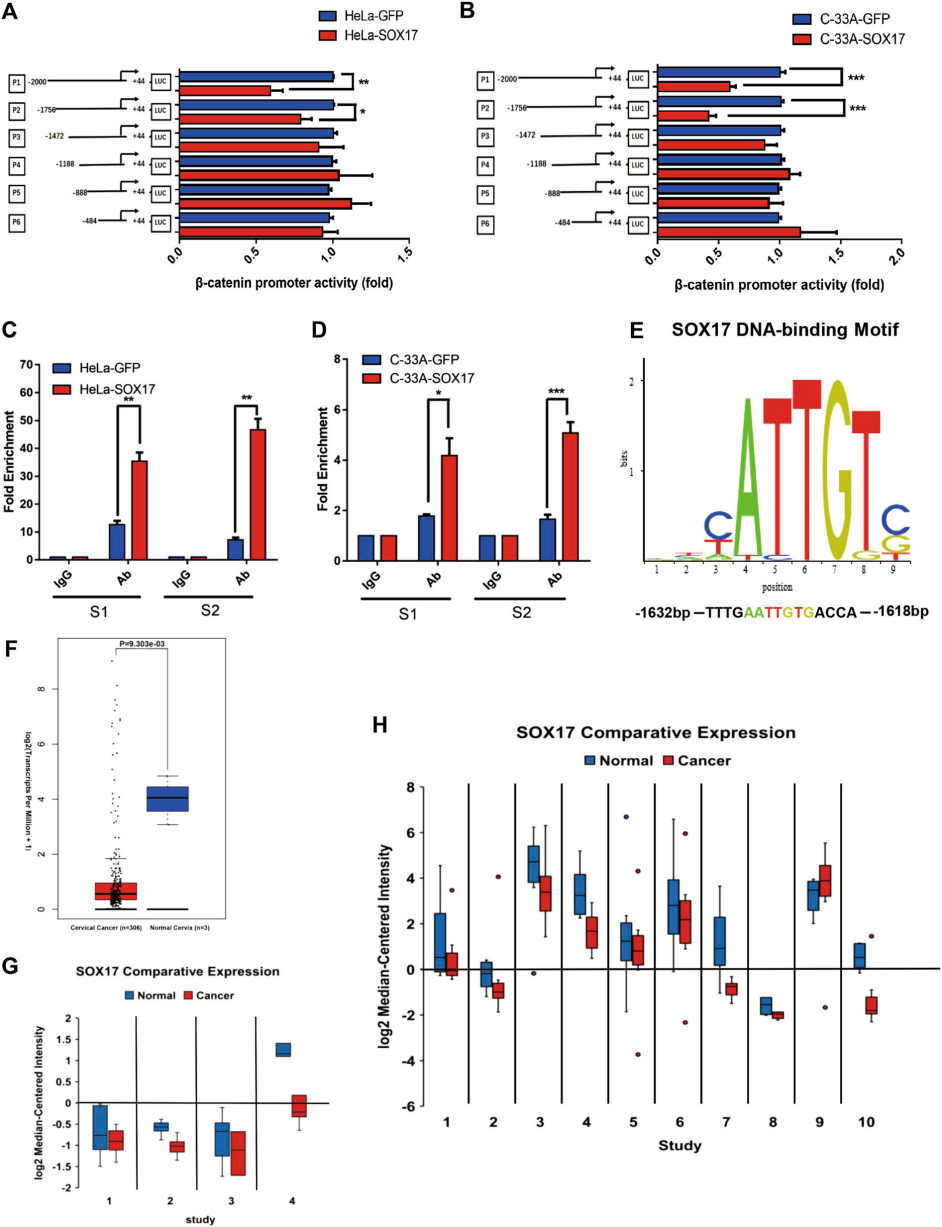
SOX17 inhibits the activity of Wnt/β-catenin pathway through directly binding to the promoter of β-catenin in cervical cancer cells. **a**, **b** The β-catenin promoter structure was constructed and luciferase activity relative to Renilla control was measured in HeLa-SOX17 and C-33A-SOX17 cells. **c**, **d** The qChIP assay is shown in the HeLa-SOX17 and C-33A-SOX17 cells immunoprecipitation by SOX17 antibody and IgG antibody (as the negative control). **e** An experimentally defined transcription factor binding sites of SOX17 was found in the JASPAR CORE database. **f** Expression of SOX17 in patients with cervical cancer compared to normal cervix tissue in TCGA database. Normal cervix = 13; cervical cancer = 306. Line in the center of the rectangle represents the median, top edge of the rectangle represents the third quartile, bottom edge of the rectangle represents the first quartile, top whisker represents the maximum and bottom whisker represents the minimum. *P* values = 0.303E-03. **g** Meta-analysis showing SOX17 downregulation in cervical cancer compared to normal cervix tissue Left to right: Zhai Cervix, Pyeon Multi-cancer, Scotto Cervix, Biewenga Cervix. Individual study sets are identified in Table S3. **h** Meta-analysis showing SOX17 downregulation in cancer compared with normal tissue. Left to right: Lee Bladder, TCGA Brain, TCGA Breast, TCGA Colorectal, Cui Gastric, Chen Liver, Hou Lung, Agnelli Myeloma, TCGA Ovarian, Barretina Sarcoma. Individual study sets are identified in Table S3. The data were shown as the mean ± SD of three independent experiments. *p *<* 0.05, **p *<* 0.01, ***p *<* 0.001

Our results above demonstrated that SOX17 inhibited the activity of Wnt/β-catenin signaling pathway through trans-suppressing the key molecule, β-catenin. Therefore, a dual-luciferase reporter assay was performed to identify if SOX17 directly bind the promoter of β-catenin in cervical cancer cells. The results showed that the transcriptional activity of cyclin D1 and c-Myc were not modulated in SOX17-overexpressing HeLa, SiHa and C-33A cell lines (Figure S4A, *p* > 0.05; Figure S4B, *p* > 0.05). Next, six luciferase reporters were constructed containing β-catenin promoter fragments with different deletions between −2000 and +44 upstream of transcriptional start site. As shown in Fig. 6a, b, the luciferase activities of P1 (−2000 to +44) and P2 (−1756 to +44) promoters were significantly decreased by SOX17 overexpression (Fig. 6a, *p* < 0.05, *p* < 0.01; Fig. 6b, *p* < 0.001, *p* < 0.001). In the other promoter regions, including P3 (−1472 to +44), P4 (−1188 to +44), P5 (−888 to +44) and P6 (−484 to +44), the luciferase activities failed to show significant differences by SOX17 overexpression (*p* > 0.05). Simultaneously, the similar results were observed in SW480 cell (Figure S4C, *p* > 0.05). These results showed that the sequence between the nucleotides −1756 and −1473 in the β-catenin promoter may contain the SOX17-binding site.

Next, quantitative ChIP (qChIP) assays were performed to identify the chromatin occupancy of SOX17 on β-catenin promoter at the specific region (−1756 and −1473) in SOX17-overexpressing cells in vivo. RT- PCR using specific S1(−1756 bp to −1610 bp) and S2 (−1614 bp to −1463 bp) primers was performed to amplify the binding region. As shown in Fig. 6c, d, enrichment of SOX17 was observed, implying that SOX17 specifically recognized and bind to S1 and S2 in the β-catenin promoter region in SOX17-overexpressing (Fig. 6c, *p* < 0.01, *p* < 0.01; Fig. 6d, *p* < 0.05, *p* < 0.001). The increased occupancy of SOX17 on S1 and S2 region of β-catenin promoter were also observed in the SW480 cell (Figure S4D, *p* < 0.01, *p* < 0.01).

As shown in Fig. 6e, an experimentally defined transcription factor binding site of SOX17, 5′-ATTGT-3′, was found in the JASPAR CORE database (http://jaspar.genereg.net/). 5′-ATTGT-3′ was located between the nucleotides −1632 and −1618, in the S1 region of  P2 promoter. However, the enrichment of SOX17 was higher in S2 than S1 (Fig. 6c, d), indicating that SOX17 might recognize and bind to the 5′-ATTGT-3′ site and/or other unknown site between the nucleotides −1756 and −1473 in the β-catenin promoter region.

To identify the clinical implications of SOX17, the expression of SOX17 was analyzed in TCGA database. The results showed that SOX17 expression was significantly reduced in cervical cancer (Fig. 6f, *p* < 0.001). A meta-analysis was performed across four studies of cervical cancer in ONCOMINE^TM^ database (Fig. 6g, left to right: Zhai Cervix^30^, Pyeon Multi-cancer^31^, Scotto Cervix^32^, Biewenga Cervix^33^). Table S3 illustrated a significant down-expression of SOX17 in cervical cancer compared to the normal cervix (*p* = 0.005). Moreover, a total of 365 studies on various cancer types were retrieved from the Oncomine^TM^ database (Figure S4E), of which 46 studies showed SOX17 low expression in cancer tissue, including breast cancer, colorectal cancer, lung cancer, sarcoma, cervical cancer, etc. 14 studies showed SOX17 was overexpressed, such as esophageal cancer and ovarian cancer, etc. Furthermore, a meta-analysis of the SOX17 expression level in cancer vs. normal across ten datasets was summarized in Fig. 6h (left to right: Lee Bladder^34^, TCGA Brain, TCGA Breast, TCGA Colorectal, Cui Gastric^35^, Chen Liver^36^, Hou Lung^37^, Agnelli Myeloma^38^, TCGA Ovarian, Barretina Sarcoma^39^). A systematic down-expression of SOX17 in multiple cancer types was observed in across the ten datasets interrogated (Table S4, *p* = 0.002).

Taken together, our findings suggested that SOX17 inhibited the tumor formation, proliferation and activity of the Wnt/β-catenin signaling pathway by directly binding to the region between the −1756 and −1473 nucleotides in the β-catenin promoter in cervical cancer cells.

## Discussion

SOX17 has been reported to be involved in definitive endoderm formation^41–43^, hematopoietic stem cells maintenance^15^, human primordial germ cell fate regulation^16^ and tumorigenesis^22^. In pancreatic tumors and glioblastomas, SOX17 has been established as a key regulator of inducing tumorigenesis and promoting tumor angiogenesis^44,45^. However, in colorectal cancer^18^, hepatocellular carcinoma^19^, gastric cancer^20^, cholangiocarcinoma^22^, breast cancer^17^, and endometrial cancer^23^, SOX17 acts as a tumor suppressor. In cervical cancer, five studies have reported the function of SOX17^24,26,46–48^. However, these studies mainly reported the phenomenon of high methylation in SOX17 promoter. Whether SOX17 participates in the process of initiation and progression of cervical cancer and how it contributes to the cervical carcinogenesis remain obscure. In the present study, IHC and western blot analysis revealed low expression of SOX17 expression in cervical cancer and the MSP assays showed high methylation of SOX17 promoter in cervical cancer cell lines, indicating that SOX17 might suppress the progression of cervical cancer. These results were in consistence with other studies in cervical cancer tissue^24,26,46–48^. Next, SOX17 was found to suppress tumor formation by inhibiting cell proliferation in vivo and in vitro through tumor xenograft, cell growth and cell viability assays. Furthermore, cell cycle analysis determined SOX17 induced cell cycle arrest at the cell transition from G_0_/G_1_ phase to S phase. This is the first study identified the SOX17 inhibited the proliferation and tumor formation by arresting the cells at cell cycle transition from G_0_/G_1_ phase to S phase in cervical cancer, in consistence with the results of another study in human oligodendroglioma^49^.

Several studies demonstrated that SOX17 inhibited the carcinogenesis by suppressing activity of the Wnt/β-catenin signaling pathway^18,19,49–54^. However, the majority of the studies did not elucidate the mechanism of SOX17 suppressing activity of Wnt/β-catenin signaling pathway^18,19,51^. Sinner et al^55^. and Chen et al^49^. reported the specific mechanism of SOX17 repressing the Wnt/β-catenin signaling pathway at the protein level in colon carcinoma and oligodendroglioma, respectively. They found that SOX17 interacted with both TCF4 and β-catenin proteins and formed a complex, mediating degradation of β-catenin and TCF4 by the proteasome independent of GSK3 activity.

In cervical cancer, Chen et al^24^. and van der Meide et al^46^. found that SOX17, together with some antagonists of WNT/β-catenin signaling pathway were highly methylated, implying an antagonism function of SOX17 in WNT/β-catenin signaling pathway. In the present study, the TOP/FOP flash luciferase reporter assay revealed that the Wnt/β-catenin signaling activity was attenuated by SOX17 in cervical cancer. Moreover, the expression of β-catenin, cyclin D1 and c-Myc, the key molecules of the Wnt/β-catenin pathway were attenuated by SOX17 at both mRNA and protein level. It seems the expression of β-catenin in microarray and RT-PCR analysis lacks concordance. It might be because the gene expression is affected by some factors, such as external environment, processing method, sampling time, etc. Also, the gene probes of microarray may detect a different region from that real-time PCR primers amplified. Next, the recombinant β-catenin plasmid was transiently transfected in the SOX17-overexpressing cells and the results showed that the proliferation suppression and cell cycle arrest of cervical cancer cells by SOX17 were rescued. Moreover, the mRNA and protein expression of cyclin D1 and c-Myc repressed by SOX17 were also elevated. Moreover, the negative correlation of SOX17 and β-catenin expression was observed in the clinical cervical cancer specimen. These results revealed that β-catenin might be the key molecule, by which SOX17 attenuate the activity of Wnt/β-catenin signaling pathway.

To further explore if SOX17 could directly trans-suppress the transcription of β-catenin, the dual-luciferase assays were conducted and revealed that SOX17 directly bind to the promoter of β-catenin, instead of cyclin D1 or c-Myc, thus deactivate the β-catenin promoter. The qCHIP assays further confirmed SOX17 directly bind to the region between −1756 and −1473 of the β-catenin promoter. The predicted binding site of 5′-ATGCG-3′ was located between the nucleotides −1632 and −1618, in the S1 region of P2 promoter. However, there was also a higher enrichment of SOX17 in S2 region, indicating that there might be another site that SOX17 recognize and bind. Moreover, these phenomena were also validated in human colon cancer cell. Our study is the first study that elucidated the specific region of β-catenin promoter, to which SOX17 directly bind to trans-suppress the β-catenin, resulting in inactivation of the Wnt/β-catenin signaling pathway.

**Fig. 7:**
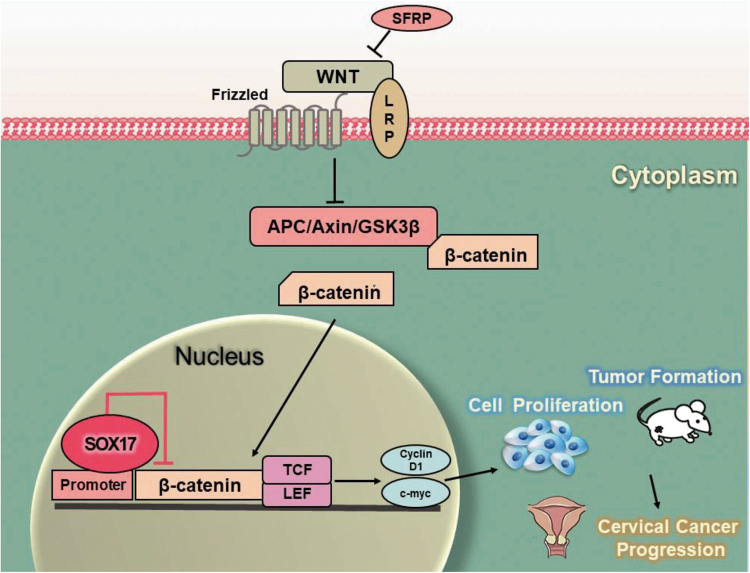
Proposed model of the SOX17-mediated disruption of Wnt/β-catenin signaling. In cervical cancer, several Wnt/β-catenin signaling antagonists including SOX17 revealed low expression because of hypermethylation. SOX17 could recognize and bind to the β-catenin promoter region as a transcription repressor and reduce the accumulation of nuclear β-catenin, leading to the down-regulation of target genes, such as cyclin D1 and c-myc. Consequently, the activity of Wnt/β-catenin signaling pathway was attenuated, resulting in cancer cell proliferation and tumor formation inhibition in cervical cancer cells

Above all, we demonstrated that SOX17 directly bind to the β-catenin promoter at the region between −1756 and −1473 and trans-suppressed β-catenin, leading to the down-regulation of β-catenin target genes, such as cyclin D1 and c-Myc. Consequently, the activity of Wnt/β-catenin signaling pathway was attenuated, resulting in the inhibition of cell proliferation and tumor formation in cervical cancer cells (Fig. 7).

In conclusion, our findings firstly demonstrated that SOX17 expression was down-regulated in cervical cancer tissues, SOX17 inhibited the tumor formation, proliferation and suppressed the activity of Wnt/β-catenin signaling pathway by directly binding to the β-catenin promoter and trans-suppressing β-catenin in cervical cancer cells.

## Electronic supplementary material

Figure S1

Figure S2

Figure S3

Figure S4

Table S1

Table S2

Table S3

Table S4

Supplementary figure legends
