# Supplementary figures and images for: SOX17 restrains proliferation and tumor formation by down-regulating activity of the Wnt/β-catenin signaling pathway via trans-suppressing β-catenin in cervical cancer
Source: Cell Death Dis. 2018 Jul 3;9(7):741. doi: 10.1038/s41419-018-0782-8 (PMC6030085; doi:10.1038/s41419-018-0782-8)

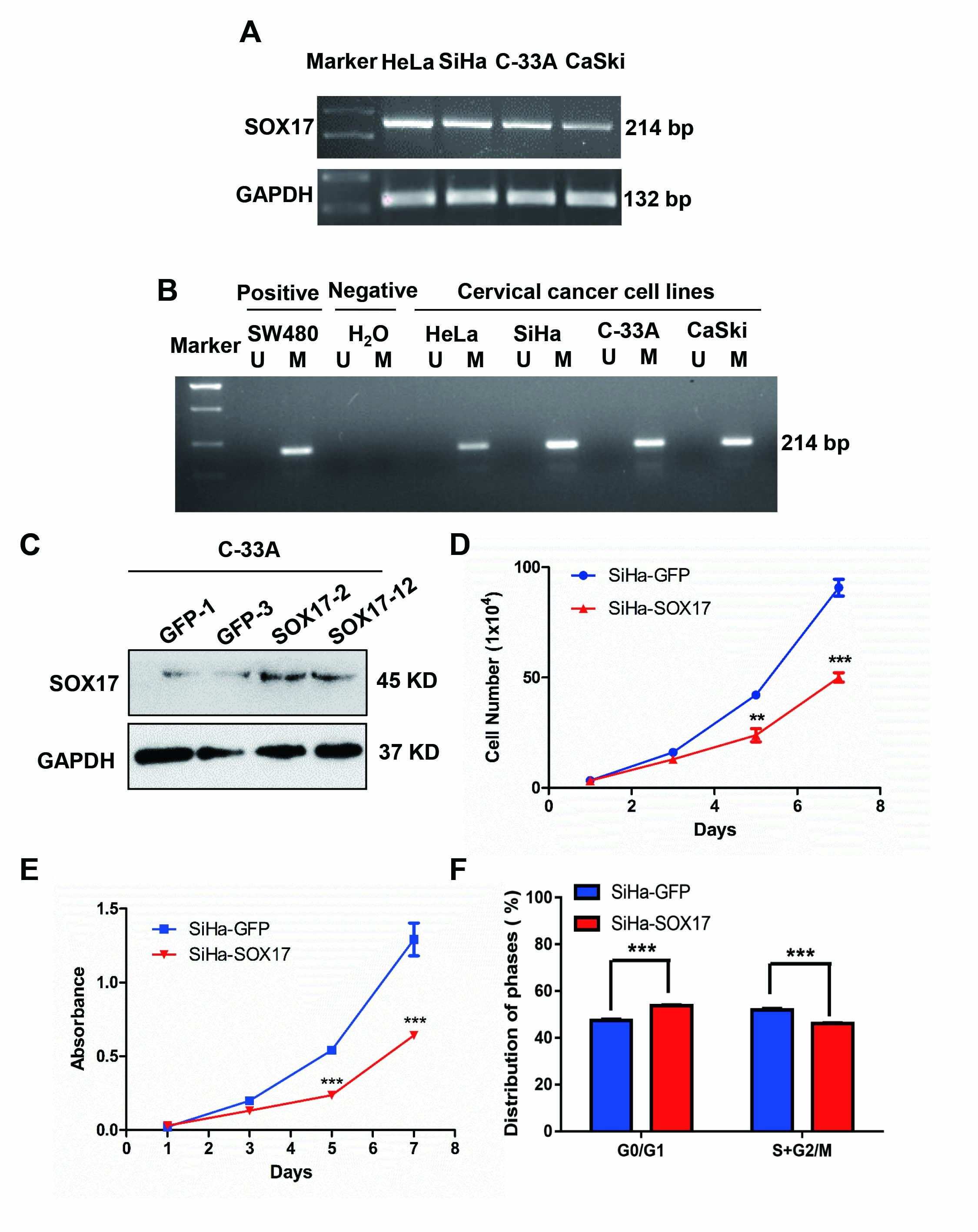

Supplement: Supplementary file 1 — Figure S1 [file 41419_2018_782_MOESM1_ESM.jpg]

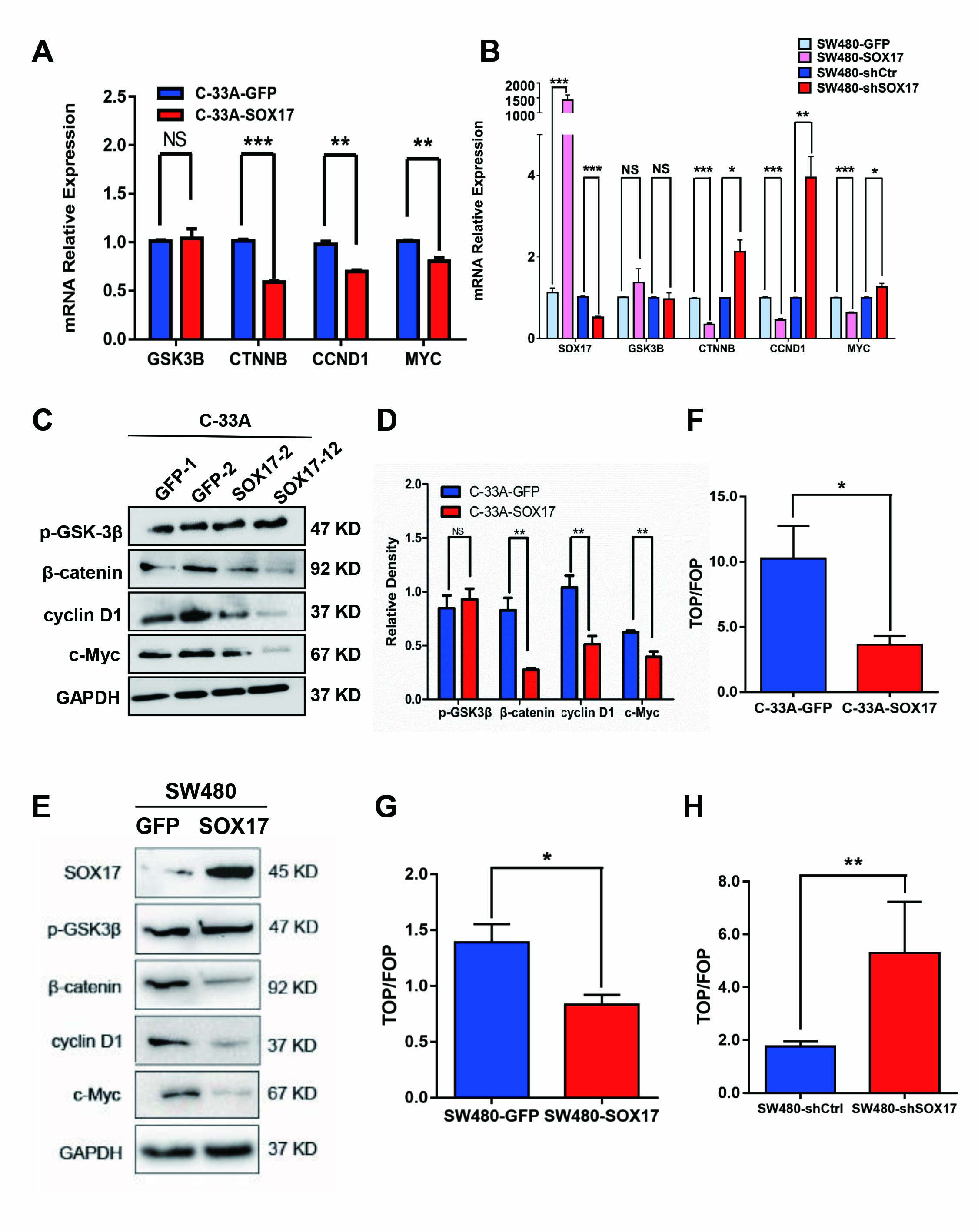

Supplement: Supplementary file 2 — Figure S2 [file 41419_2018_782_MOESM2_ESM.jpg]

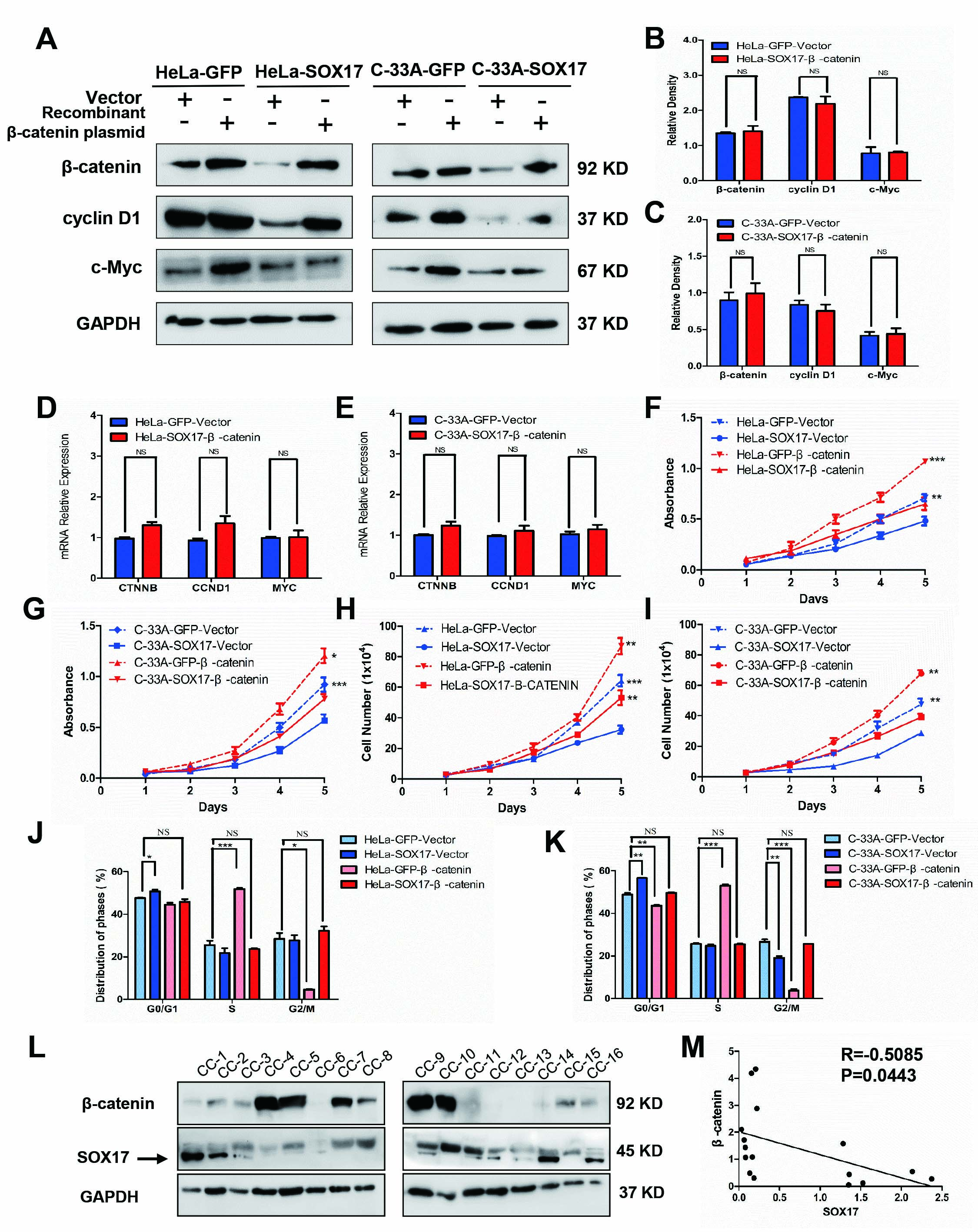

Supplement: Supplementary file 3 — Figure S3 [file 41419_2018_782_MOESM3_ESM.jpg]

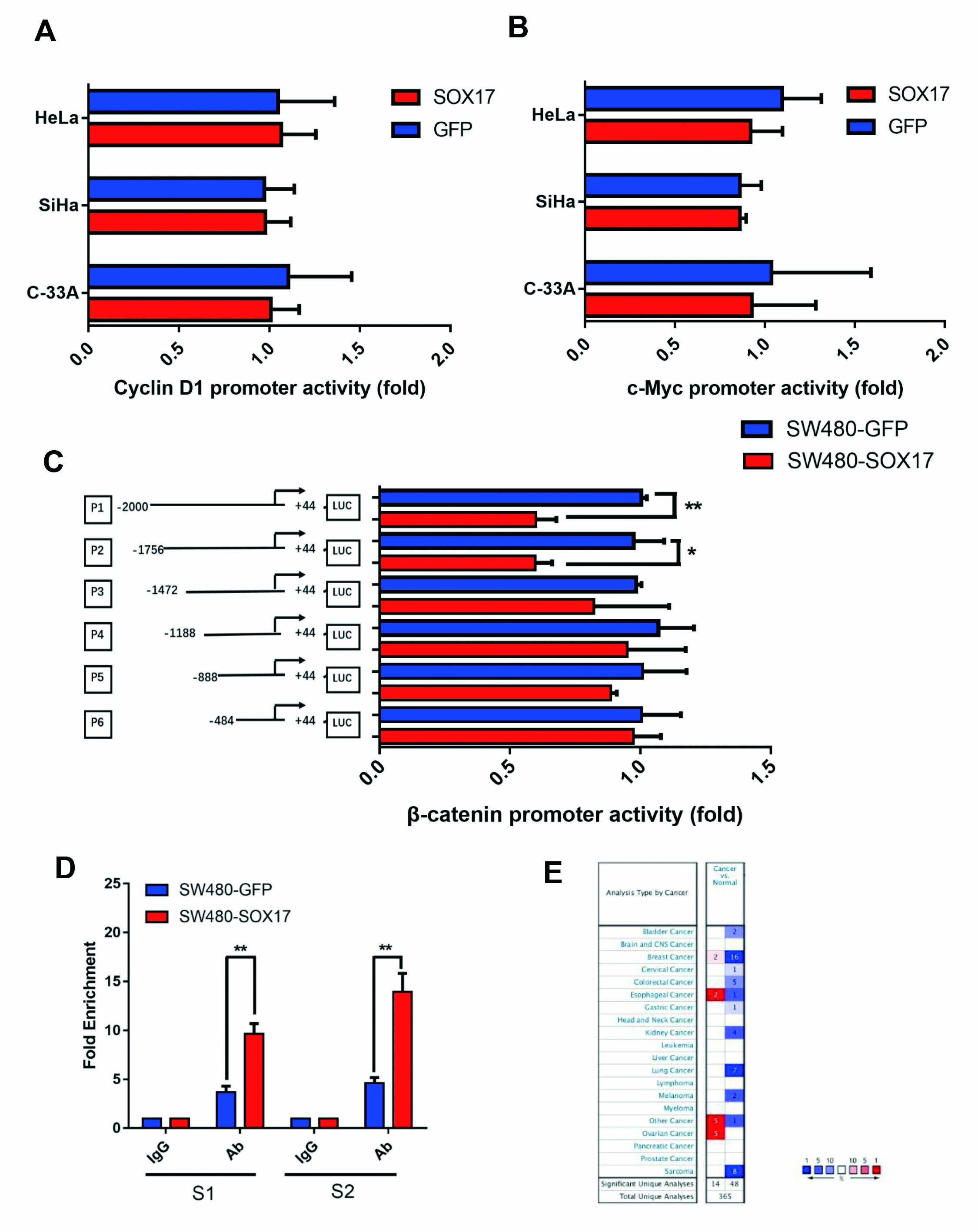

Supplement: Supplementary file 4 — Figure S4 [file 41419_2018_782_MOESM4_ESM.jpg]
