# Supplementary material for: SOX17 restrains proliferation and tumor formation by down-regulating activity of the Wnt/β-catenin signaling pathway via trans-suppressing β-catenin in cervical cancer
Source: Cell Death Dis. 2018 Jul 3;9(7):741. doi: 10.1038/s41419-018-0782-8 (PMC6030085; doi:10.1038/s41419-018-0782-8)
Supplement: Supplementary file 5 — Table S1 [file 41419_2018_782_MOESM5_ESM.doc]

**Supplementary Table S1: SOX17 expression levels in different tissue specimen**

| **Specimens** | **Total** | **SOX17 Staining** | | *P* |
| --- | --- | --- | --- | --- |
|  |  | **Negative, No. (%)** | **Positive, No. (%)** |  |
| Normal | 31 | 2（6.4） | 29（93.6） |  |
| HSIL | 20 | 4（20.0） | 16（80.0） | < 0.05a |
| Carcinoma | 67 | 33（49.3） | 34（50.7） | < 0.05a |

Abbreviation: SOX17

Pearson 2-tailed chi-square test was used to determine the statistical significance of the level of expression of SOX17 in different tissue specimens.

aNormal cervix versus high-grade squamous intraepithelial lesion.

bNormal cervix versus carcinoma.
