# Supplementary material for: SOX17 restrains proliferation and tumor formation by down-regulating activity of the Wnt/β-catenin signaling pathway via trans-suppressing β-catenin in cervical cancer
Source: Cell Death Dis. 2018 Jul 3;9(7):741. doi: 10.1038/s41419-018-0782-8 (PMC6030085; doi:10.1038/s41419-018-0782-8)
Supplement: Supplementary file 6 — Table S2 [file 41419_2018_782_MOESM6_ESM.doc]

**Supplementary Table S2: The list of primer sequences that used in this study**

| **Recombinant Vector Construction** | |
| --- | --- |
| SOX17-CDS | F: GAAGATCTGCCACCATGAGCAGCCCGGATGCG |
|  | R: GCGCCGGAATTCTCACACGTCAGGATAGTTGCAG |
| CTNNB-CDS | F: ATGGCTACTCAAGCTGATTTGATG |
|  | R: TTACAGGTCAGTATCAAACCAGGC |
| shSOX17-1477 | F:CACCGGCCAGAAGCAGTGTTACACATTCAAGAGATGTGTAACACTGCTTCTGGCCTTTTTTG |
|  | R:GATCCAAAAAAGGCCAGAAGCAGTGTTACACATCTCTTGAATGTGAACACTGCTTCTGGCC |
| shSOX17-1602 | F:CACCGTCTGCCACTTGAACAGTTTGTTCAAGAGACAAACTGTTCAAGTGGCAGACTTTTTTG |
|  | R:GATCCAAAAAAGTCTGCCACTTGAACAGTTTGTCTCTTGAACAAACTGTTCAAGTGGCAGAC |
| shCtrl | F:CACCGTTCTCCGAACGTGTCACGTTTCAAGAGAACGTGACACGTTCGGAGAATTTTTTG |
|  | R:GATCCAAAAAATTCTCCGAACGTCTCACGTTCTCTTGAAACGTGACACGTTCGGAGAAC |
| **RT-PCR** | |
| SOX17 | F: AGTGACGACCAGAGCCAGAC |
|  | R: CCTTAGCCCACACCATGAAA |
| GSK3B | F: GGCAGCATGAAAGTTAGCAGA |
|  | R: GGCGACCAGTTCTCCTGAATC |
| CTNNB | F: TCTGAGGACAAGCCACAAGATTACA |
|  | R: TGGGCACCAATATCAAGTCCAA |
| CDK4 | F: ATGTTGTCCGGCTGATGGA |
|  | R: CACCAGGGTTACCTTGATCTCC |
| CCND1 | F: AAACAGATCATCCGCAAACAC |
|  | R: GTTGGGGCTCCTCAGGTTC |
| MYC | F: CCTGGTGCTCCATGAGGAGA |
|  | R: TCCAGCAGAAGGTGATCCAGAC |
| GAPDH | F: GCACCGTCAAGGCTGAGAAC |
|  | R: TGGTGAAGACGCCAGTGGA |
| **Luciferase Assays** | |
| P1 (-2000 bp-+44 bp) | F: GTTTACGGTGTCAGTAGGGAT |
| P2 (-1756 bp-+44 bp) | F: GCATATTCCTTAATTCTGCAAATGAC |
| P3 (-1472 bp-+44 bp) | F: CGGCAGTTGGCATTACCACT |
| P4 (-1188 bp-+44 bp) | F: GAGAAATCGATCATACTTGTTGCAG |
| P5 (-888 bp -+44 bp) | F: GCTCTGGAGCTAATCCATTTCC |
| P6 (-484 bp -+44 bp) | F: AACTTCCGCCCTCCCAGGAC |
|  | R: GCTCTTATAAGTCGCGCAGAAG |
| Cyclin D1 promoter region (-2024 bp-+200 bp) | F: CTAGCTAGCCGGGAAATCAACGAAGTTCCTAGTC |
|  | R: CCCAAGCTTAGAAACACCACGGCAAACTTCAAAG |
| c-Myc Promoter region (-1975 bp-+249 bp) | F: CTAGCTAGCGCGATGATCTCTGCTGCCAGTAGAG |
|  | R: CCCAAGCTTTTCCACTACCCGAAAAAAATCCAGC |
| **Chromatin Immunoprecipitation Assay (ChIP)** | |
| S1 (-1756 bp—-1610 bp) | F: GCATATTCCTTAATTCTGCAAATG |
|  | R: ATTGGTTGTGGTCACAATTC |
| S2 (-1614 bp—-1463 bp) | F: CCACAACCAATAGCTACACTTAGAT |
|  | R: CCAACTGCCGTCTGTCACC |
| 3’UTR | F: TGGGTAGGGTAAATCAGTAAGAGGT |
|  | R: GAAGCATCGTATCACAGCAGGTT |
| **Methylation-Specific PCR (MSP)** | |
| SOX17 methylated | F: GGAGATTCGCGTAGTTTTCG |
|  | R: AACCCGACCATCACCGCG |
| SOX17 unmethylated | F: GGAGATTTGTGTAGTTTTTG |
|  | R: AACCCAACCATCACCACA |
