# Supplementary material for: SOX17 restrains proliferation and tumor formation by down-regulating activity of the Wnt/β-catenin signaling pathway via trans-suppressing β-catenin in cervical cancer
Source: Cell Death Dis. 2018 Jul 3;9(7):741. doi: 10.1038/s41419-018-0782-8 (PMC6030085; doi:10.1038/s41419-018-0782-8)
Supplement: Supplementary file 7 — Table S3 [file 41419_2018_782_MOESM7_ESM.docx]

**Supplementary Table S3 Meta-analysis of SOX17 expression in cervical cancer**

| **No** | **Study** | **Rank** | **Top^1^ (%)** | **P-value^2^** | **Fold change** | **Normal/Cancer samples** | **Link to data** |
| --- | --- | --- | --- | --- | --- | --- | --- |
| 1 | Zhai Cervix | 5141 | 41 | 0.154 | -1.126 | 10/21 | <http://www.ncbi.nlm.nih.gov/geo/query/acc.cgi?acc=GSE7803> |
| 2 | Pyeon Multi-cancer | 1707 | 9 | 4.42E-06 | -1.242 | 8/20 | <http://www.ncbi.nlm.nih.gov/geo/query/acc.cgi?acc=GSE6791> |
| 3 | Scotto Cervix | 2508 | 20 | 0.009 | -1.449 | 25/32 | <http://www.ncbi.nlm.nih.gov/geo/query/acc.cgi?acc=GSE9750> |
| 4 | Biewenga Cervix | 2615 | 14 | 4.60E-04 | -2.265 | 5/40 | <http://www.ncbi.nlm.nih.gov/geo/query/acc.cgi?acc=GSE7410> |
|  | Total^3^ | 2561.5 | NA | 0.005 | NA | NA | NA |

1 Top refers to the top percent of the SOX17 rank out of the differential expression genes.

2 The P value of t-Test was reported as the statistical output of the original studies as presented on Oncomine.

3 The meta-analysis was performed using Oncomine (http://Oncomine.org) for cervical cancer data sets.
