# Supplementary material for: SOX17 restrains proliferation and tumor formation by down-regulating activity of the Wnt/β-catenin signaling pathway via trans-suppressing β-catenin in cervical cancer
Source: Cell Death Dis. 2018 Jul 3;9(7):741. doi: 10.1038/s41419-018-0782-8 (PMC6030085; doi:10.1038/s41419-018-0782-8)
Supplement: Supplementary file 8 — Table S4 [file 41419_2018_782_MOESM8_ESM.docx]

**Supplementary Table S4 Meta-analysis of SOX17 expression in multi-cancer**

| No | Study | Rank | Top^1^ (%) | P-value^2^ | Fold change | Normal/Cancer samples | Link to data |
| --- | --- | --- | --- | --- | --- | --- | --- |
| 1 | Lee Bladder | 1328 | 8 | 6.49E-06 | -1.778 | 68/62 | http://www.ncbi.nlm.nih.gov/geo/query/acc.cgi?acc=GSE13507 |
| 2 | TCGA Brain | 4176 | 34 | 0.005 | -1.488 | 10/542 | http://tcga-data.nci.nih.gov/tcga/ |
| 3 | TCGA Breast | 1590 | 8 | 2.94E-13 | -2.374 | 61/76 | http://tcga-data.nci.nih.gov/tcga/ |
| 4 | TCGA Colorectal | 1712 | 9 | 1.59E-09 | -3.198 | 22/22 | http://tcga-data.nci.nih.gov/tcga/ |
| 5 | Cui Gastric | 1472 | 9 | 0.004 | -1.394 | 80/80 | http://www.ncbi.nlm.nih.gov/geo/query/acc.cgi?acc=GSE27342 |
| 6 | Chen Liver | 1960 | 19 | 3.62E-04 | -1.576 | 73/100 | http://www.ncbi.nlm.nih.gov/geo/query/acc.cgi?acc=GSE3500 |
| 7 | Hou Lung | 116 | 1 | 3.33E-26 | -4.393 | 65/27 | http://www.ncbi.nlm.nih.gov/geo/query/acc.cgi?acc=GSE19188 |
| 8 | Agnelli Myeloma | 2329 | 19 | 0.036 | -1.308 | 5/9 | http://www.ncbi.nlm.nih.gov/geo/query/acc.cgi?acc=GSE13591 |
| 9 | TCGA Ovarian | 9046 | 72 | 0.969 | 1.393 | 8/586 | http://tcga-data.nci.nih.gov/tcga/ |
| 10 | Barretina Sarcoma | 85 | 1 | 4.87E-11 | -4.463 | 9/26 | http://www.ncbi.nlm.nih.gov/geo/query/acc.cgi?acc=GSE21122 |
|  | Total^3^ | 1531 |  | 0.002 |  |  |  |

1 Top refers to the top percent of the SOX17 rank out of the differential expression genes.

2 The P value of t-Test was reported as the statistical output of the original studies as presented on Oncomine.

3 The meta-analysis was performed using Oncomine (http://Oncomine.org) for 10 types of cancer data sets.
