# Supplementary material for: SOX17 restrains proliferation and tumor formation by down-regulating activity of the Wnt/β-catenin signaling pathway via trans-suppressing β-catenin in cervical cancer
Source: Cell Death Dis. 2018 Jul 3;9(7):741. doi: 10.1038/s41419-018-0782-8 (PMC6030085; doi:10.1038/s41419-018-0782-8)
Supplement: Supplementary file 9 — Supplementary figure legends [file 41419_2018_782_MOESM9_ESM.docx]

**SFigure 1. Methylation of the sox17 promoter in cervical cancer cell lines and the inhibitory functions of SOX17 on proliferation of C-33A-SOX117 cells and SiHa-SOX17 cells.** (A). SOX17 expression in the cervical cancer cell-lines was detected by RT-PCR. (B). MS-PCR for a region of the SOX17 promoter in the 4 cervical cancer cell lines. A methylated band was amplified in SiHa and C33A cells. SW480 cell was included as a positive control for the methylated (M) and unmethylated (U) primers. (C). Stably transfected cell lines were identified by western blotting: C33A-GFP and C33A-SOX17 cells. (D). The proliferation was detected using growth curves in SiHa-GFP and SiHa -SOX17 cells. (E). The viability was detected by the MTT assay in SiHa -GFP and SiHa -SOX17 cells. (F). The cell cycles of SiHa-GFP and SiHa-SOX17 cells were analyzed using flow cytometry and a quantitative analysis of the cell cycle is shown.

**SFigure 2. SOX17 induces cell cycle arrest by trans-suppressing the Wnt/β-catenin pathway in C-33A cells and SW480 cells.**

(A). Real-time PCR analysis is shown for the mRNA levels of Wnt/β-catenin pathway key genes in C-33A-SOX17 cells. (B) Real-time PCR analysis is shown for the mRNA levels of Wnt/β-catenin pathway key genes in SW480 cell. (C). The expression of Wnt/β-catenin pathway key proteins in C-33A-SOX17 cells were determined by western blot. (D). The quantitative analysis of western blot was shown. (E). The expression of Wnt/β-catenin pathway key proteins in SW480-SOX17 cells were determined by western blot. (F). C-33A-SOX17 cells, SW480-SOX17 cells (G) and SW480-shSOX17 cells were transfected with the TOP/FOP-Flash reporter plasmid, and the reporter activities were detected 48h after transfection by a luciferase assay. Data represent mean±SD of triplicate experiments and statistical analyzed with student’s t-test. * *p<0.05, ** p<0.01, *** p<0.001.*

**SFigure 3. Up-regulating β-catenin attenuates the proliferation suppression of SOX17 in cervical cancer cells.** (A)The expressions of β-catenin, c-myc and cyclinD1 were detected by western blot in β-catenin transiently transfected HeLa-SOX17 and C-33A-SOX17 cells. (B and C) The quantitative analyses of western blot were shown. (D and E) The transcript level of GSK3B, CCND1 and C-MYC in β-catenin transiently transfected HeLa-SOX17 and C-33A-SOX17 cells were determined by Real-time PCR. The proliferation and viability of β-catenin transiently transfected HeLa-SOX17 and C-33A-SOX17 cells were detected by growth curves (F and G) and MTT assay (H and I). (J and K) The quantitative analysis of the cell cycle of β-catenin transiently transfected HeLa-SOX17 and C-33A-SOX17 cells is shown. (L and M) The expression of SOX17 and β-catenin was detected by Western blot and the correlation was analyzed. The data were shown as the mean ± SD of three independent experiments. **p < 0.05, **p < 0.01, ***p < 0.001*.

**SFigure 4. SOX17 inhibits the activity of Wnt/β-catenin pathway through directly binding to the promoter of β-catenin not cyclin D1 or c-Myc in cervical cancer cells and colon cancer cell.**

The cyclin D1(A) and c-Myc (B) full-length promoter structure was constructed and luciferase activity relative to Renilla control was measured in HeLa-SOX17, C-33A-SOX17 cells and SiHa cells, respectively. (C) The β-catenin promoter structure was constructed and luciferase activity relative to Renilla control was measured in SW480 cells. (D) The qChIP assay is shown in the SW480-SOX17 cells Immunoprecipitation by SOX17 antibody and IgG antibody (as the negative control). (E). The summary of the SOX17 expression of different cancer types in Oncomine database. Different colors refer to different gene rank percentile. **p < 0.05, **p < 0.01*.
